# SH3 Domain-Mediated Recruitment of Host Cell Amphiphysins by Alphavirus nsP3 Promotes Viral RNA Replication

**DOI:** 10.1371/journal.ppat.1002383

**Published:** 2011-11-17

**Authors:** Maarit Neuvonen, Arunas Kazlauskas, Miika Martikainen, Ari Hinkkanen, Tero Ahola, Kalle Saksela

**Affiliations:** 1 Institute of Biotechnology, University of Helsinki, Helsinki, Finland; 2 Department of Virology, Haartman Institute, University of Helsinki and Helsinki University Central Hospital, Helsinki, Finland; 3 A. I. Virtanen Institute, Department of Biotechnology and Molecular Medicine, University of Eastern Finland and the Cancer Center of Eastern Finland, Kuopio, Finland; Institut Pasteur, France

## Abstract

Among the four non-structural proteins of alphaviruses the function of nsP3 is the least well understood. NsP3 is a component of the viral replication complex, and composed of a conserved aminoterminal macro domain implicated in viral RNA synthesis, and a poorly conserved carboxyterminal region. Despite the lack of overall homology we noted a carboxyterminal proline-rich sequence motif shared by many alphaviral nsP3 proteins, and found it to serve as a preferred target site for the Src-homology 3 (SH3) domains of amphiphysin-1 and -2. Nsp3 proteins of Semliki Forest (SFV), Sindbis (SINV), and Chikungunya viruses all showed avid and SH3-dependent binding to amphiphysins. Upon alphavirus infection the intracellular distribution of amphiphysin was dramatically altered and colocalized with nsP3. Mutations in nsP3 disrupting the amphiphysin SH3 binding motif as well as RNAi-mediated silencing of amphiphysin-2 expression resulted in impaired viral RNA replication in HeLa cells infected with SINV or SFV. Infection of Balb/c mice with SFV carrying an SH3 binding-defective nsP3 was associated with significantly decreased mortality. These data establish SH3 domain-mediated binding of nsP3 with amphiphysin as an important host cell interaction promoting alphavirus replication.

## Author Summary

The genus Alphavirus contains 29 known species that are transmitted by arthropods and include many important pathogens, such as Chikungunya virus (CHKV), which during the past decade has re-emerged to cause massive epidemics of febrile arthralgia around the Indian Ocean. The role of the alphaviral non-structural protein 3 (nsP3) has been linked to RNA replication and disease pathogenesis, but its molecular functions have remained elusive. Here we show that the nsP3s of CHKV as well as Sindbis and Semliki Forest viruses use a conserved proline-rich motif to interact with the Src-homology-3 (SH3) domain of host cell amphiphysins Amph1 and BIN1/Amph2, two adaptor proteins prominently involved in cellular membrane dynamics. We observed a striking re-localization of amphiphysin to alphaviral replication complexes in infected cells, and found that disruption of the amphiphysin SH3 binding motif in nsP3 strongly suppressed virus replication in vitro and attenuated Semliki Forest virus in infected mice. Thus, we conclude that amphiphysins are novel and important host cell factors involved in supporting alphavirus replication.

## Introduction

The genus Alphavirus (family *Togaviridae*) includes some 30 known members. The alphaviruses are enveloped positive-strand RNA viruses with a 5′ capped and 3′ polyadenylated genome of approximately 11.5 kb. Most alphaviruses are mosquito-borne viruses, and some are capable of causing serious disease in humans and domestic animals [1], [2]. On the American continents, Venezuelan, Western, and Eastern equine encephalitis viruses occasionally cause epidemics in horses, which can also spill over to infect humans with potentially lethal consequences. In contrast, Old World alphaviruses, including Ross River virus and Sindbis virus (SINV), are associated with fever, rash and painful, debilitating arthritis, which can persist for months or even years. Most recently, starting in 2005, Chikungunya virus (CHKV) re-emerged to cause a large epidemic around the Indian Ocean, infecting approximately 10 million people [3].

Alphavirus RNA replication takes place in small membrane invaginations that protrude from the inner surface of the host cell plasma membrane and from the outer surface of endosomes and lysosomes [4]. In infected cells the endo-lysosomes are ultrastructurally altered by the viral replication complexes, and are termed cytopathic vacuoles type I (CPVs) [5]. The replication complexes contain as essential components the virus-encoded nonstructural proteins nsP1-nsP4, which arise through cleavage from a polyprotein precursor P1234. NsP1, nsP2 and nsP4 possess essential enzymatic activities of RNA capping, helicase/protease, and polymerase, respectively (for a review see [6]).

The functions of nsP3 have remained more obscure, although mutations in it affect various steps of RNA synthesis [7]. The N-terminus of nsP3 contains a structurally conserved protein domain termed the *macro* domain, which is capable of binding ADP-ribose derivatives and RNA, and also hydrolyzing ADP-ribose-1′′-phosphate [8], [9]. Although the roles of these activities in RNA replication remain to be clarified, mutations in the *macro* domain affect RNA synthesis [10]. The C-terminus of alphavirus nsP3 contains a ‘tail’ region, which varies in length between ∼150–250 amino acid residues in different alphavirus and is devoid of predicted secondary structure. Interestingly, the tail is ‘hypervariable’, showing no overall sequence conservation even between related alphaviruses. Nevertheless, the tail region has been implicated in the virulence of alphaviruses [11]. Some regions of the tail are also heavily phosphorylated on serine and threonine residues, and in Semliki Forest virus (SFV), deletion of the phosphorylated region gave rise to a virus that replicated well in cell culture but was apathogenic in mice [12].

Multiple host proteins associated with nsP3 have been identified via immunoprecipitation and mass spectrometry [13], [14], but the significance of these interactions as well as the relevant protein binding sites involved have remained uncharacterized. In addition to being present in the replication complexes in the CPVs and at the plasma membrane, a large fraction of nsP3 dissociates from the other nsPs, and is found in large cytoplasmic granules of unknown function [15]. It is thus possible that different interaction partners could be found in the replication complexes and in the cytoplasmic granules [14].

Src homology-3 (SH3) domains represent a ubiquitous family (∼300 members in the human proteome) of modular protein binding domains. SH3 domains are small (∼60 residues) globular protein units that mediate interactions between proteins that are typically involved in cell signaling, membrane trafficking, and cytoskeletal organization via binding to proline-rich target sites in their ligands [16], [17]. As first noted for the HIV-1 Nef protein [18], several pathogens also encode proteins that interact with the host cell via SH3-mediated contacts.

Prompted by the presence of conserved cluster of proline residues in the otherwise poorly conserved C-terminal tails of alphaviral nsP3 proteins, we have analyzed the potential roles of SFV, CHKV and SINV nsP3 as ligands for cellular SH3 domain-containing proteins. We discovered that all these proteins show strong SH3-mediated interactions with amphiphysin-1 and Bin1/amphiphysin-2, two related proteins prominently involved in endocytosis and membrane trafficking. Deletions or point mutations affecting the SH3 binding motifs of nsP3 abolished the interaction with amphiphysins both in transfected and in virus-infected cells. In the infected cells, amphiphysins were recruited to the sites of RNA replication, and mutations in the nsP3 SH3 binding motifs led to reduced virus replication in cell culture and attenuated pathogenicity in infected mice.

## Results

### Alphaviral nsP3 proteins are amphiphysin SH3 domain ligands

**Figure 1:**
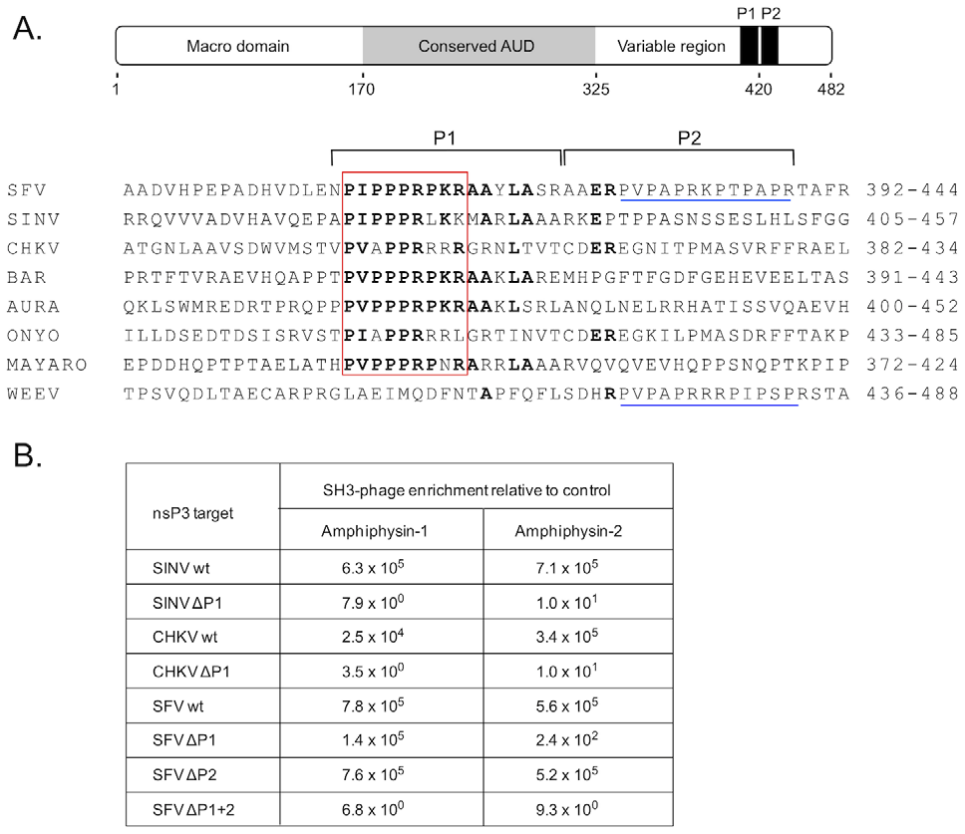
SH3 domains of amphiphysin-1 and -2 bind to conserved proline-rich regions shared by alphaviral nsP3 proteins. (**A**) Under a schematic representation of the overall structural organization of SFV nsP3 are shown carboxyterminal regions of selected alphaviral nsP3 proteins aligned based on a shared proline-rich region, dubbed the “PIPPPR motif” (boxed in red). Indicated on the right is the amino acid numbering of the aligned nsP3 regions of Semliki Forest (SFV), Sindbis (SINV), Chikungunya (CHKV), Barmah Forest (BAR), Aura, O'nyong-nyong (ONYO), Mayaro viruses, and Western equine encephalomyelitis virus (WEEV). The amino acids identical in at least four sequences are shown in bold. The regions targeted by deletions (P1 and P2) are indicated by horizontal square brackets above the sequences. Another proline-rich region found in the P2 region in the SFV and WEEV is underlined in blue. Of note, WEEV nsP3 contains only this second motif but is lacking the PIPPPR motif. (**B**) Binding of phages displaying the SH3 domain of amphiphysin-1 or -2 bind to wild-type or deletion mutants of SIN, CHKV, or SFV nsP3 proteins. The numbers indicate relative phage binding compared to a parallel mock experiment without nsP3.

The carboxyterminal halves of nsP3 proteins of alphaviruses are poorly conserved, but characterized by regions rich in proline residues (Figure 1A). The presence of arginine residues within these proline-rich clusters is a hallmark of peptide binding motifs recognized by modular protein interaction domains of the SH3 family [16], leading us to examine the possibility that nsP3 proteins might be ligands for SH3 domain-containing host cell proteins.

To address this issue we used the nsP3 proteins of SFV, SINV, and CHKV for screening of a bacteriophage library displaying on its surface a virtually complete collection of human SH3 domains. This tool was developed and validated in our laboratory a couple of years ago [19], and has since been successfully used to identify preferred SH3 partners for a number of cellular, viral, as well as bacterial proteins [20]–[25].

SFV, SINV, and CHKV nsP3 proteins all turned out to be excellent affinity reagents for panning (affinity selection) of this SH3 phage display library, and showed more than 1000-fold enrichment of phages compared to the mock protein used as a control. The identities of individual SH3 clones obtained after a single round of panning with SFV, SINV, or CHKV nsP3 were determined. In all three cases the SH3 domains of the related proteins amphiphysin-1 or -2 were the most commonly observed clones, and together constituted more than 90% (65 of the 72 identified clones) of all SH3 domains selected by SFV, SINV, and CHKV nsP3. The other seven clones represented the SH3 domains of the adapter proteins CMS/CD2AP (n = 4), CIN85 (n = 2), and SASH1 (n = 1), whose significance as nsP3 interaction partners remains to be addressed.

In accordance with their prominent selection from the human SH3 library, use of individual homogenous phage preparations displaying SH3 domains of amphiphysin-1 or -2 confirmed their capacity for robust binding to SFV, SINV, and CHKV nsP3 (Figure 1B). The same experimental approach was also used to confirm that the conserved proline-rich regions shared by these nsP3 proteins were responsible for their amphiphysin SH3 binding. Indeed, a 17-amino acid deletion spanning the conserved proline-rich region (ΔP1) resulted in a 4-log drop in binding to the SINV and CHKV nsP3 proteins (Figure 1B). Unexpectedly, in SFV nsP3 this deletion caused a less pronounced decrease in SH3 binding, especially in the case of amphiphysin-1. This observation directed our attention to a second proline-rich region (P2 in Figure 1A) in SFV nsP3 immediately adjacent to the deleted P1 region. When the deletion in SFV nsP3 was extended to include P2 (ΔP1+2 in Figure 1B) a loss of amphiphysin SH3 binding similar to that of the ΔP1 mutants of SINV and CHKV nsP3 was observed. Thus, in addition to the major amphiphysin SH3 binding motif (P1) shared by all these nsP3 proteins, SFV contains an additional site (P2) that can independently support binding to amphiphysin SH3.

**Figure 2:**
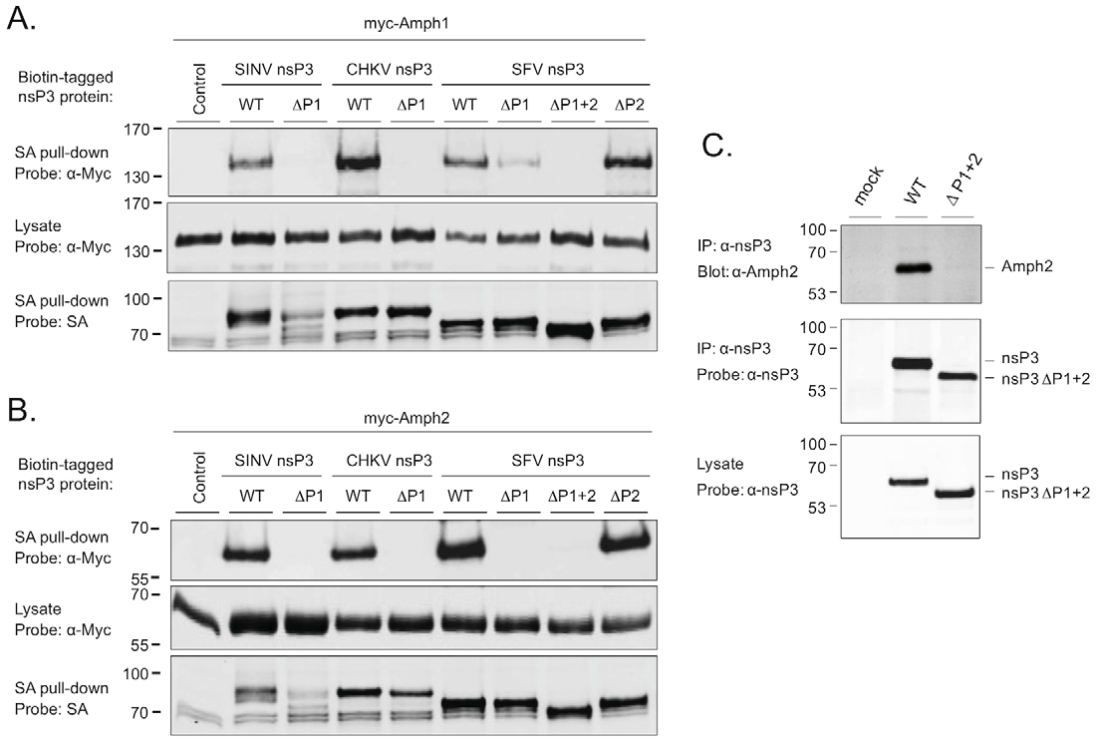
The SH3 binding site of alphaviral nsP3 proteins mediates binding to amphiphysin-1 and -2 proteins in transfected and in infected cells. Expression vectors for complete or proline rich region-deleted versions of SINV, CHKV, and SFV nsP3 expressed as fusion proteins with a biotin acceptor domain were cotransfected to 293 cells together with the Myc-tagged amphiphysin-1 (A) or amphiphysin-2 (B). Lysates of the transfected cells were subjected to a precipitation with streptavidin-coated beads, followed by Western blot analysis using labeled streptavidin or anti-Myc antibodies to detect associated nsP3 (top panels) and amphiphysin (bottom panels) proteins, respectively. To confirm uniform expression of the amphiphysin proteins in transfected cells, total lysates were also examined by anti-Myc Western blot analysis (middle panels). (C) HeLa cells were infected with wild-type SFV or a modified virus carrying the ΔP1+2 mutant of nsP3. Lysates of the infected cells were examined by anti-nsP3 Western blot analysis (bottom panel), or subjected to immunoprecipitation with anti-nsP3 antibodies followed by Western blotting analysis of the immunocomplexes using antibodies against amphiphysin-2 (top panel) or nsP3 (middle panel). A lysate prepared from mock-infected cells was included as a negative control.

To validate and examine these interactions in the context of full-length proteins expressed in human cells, we generated expression vectors for tagged versions of amphiphysin-1 and -2 (fused with a Myc epitope) as well as SFV, SINV, and CHKV nsP3 proteins (fused with a biotin acceptor domain [20]). Co-precipitation experiments revealed a robust association of all these three nsP3 proteins with both amphiphysins from lysates of transfected cells (Figures 2A and B). In agreement with our data on binding of the individual amphiphysin SH3 phage preparations (Figure 1B), deletion of the P1 region from SINV or CHKV nsP3 abolished coprecipitation with amphiphysins, whereas a combined deletion of P1 and P2 was required to completely prevent interaction of SFV nsP3 with the amphiphysins. However, in the context of full-length proteins, the role of P1 in SFV nsP3 was clearly dominant. Deletion of P2 alone had little or no effect on overall amphiphysin binding by SFV nsP3, and on its own P2 could mediate only weak co-precipitation of amphiphysin-1.

**Figure 3:**
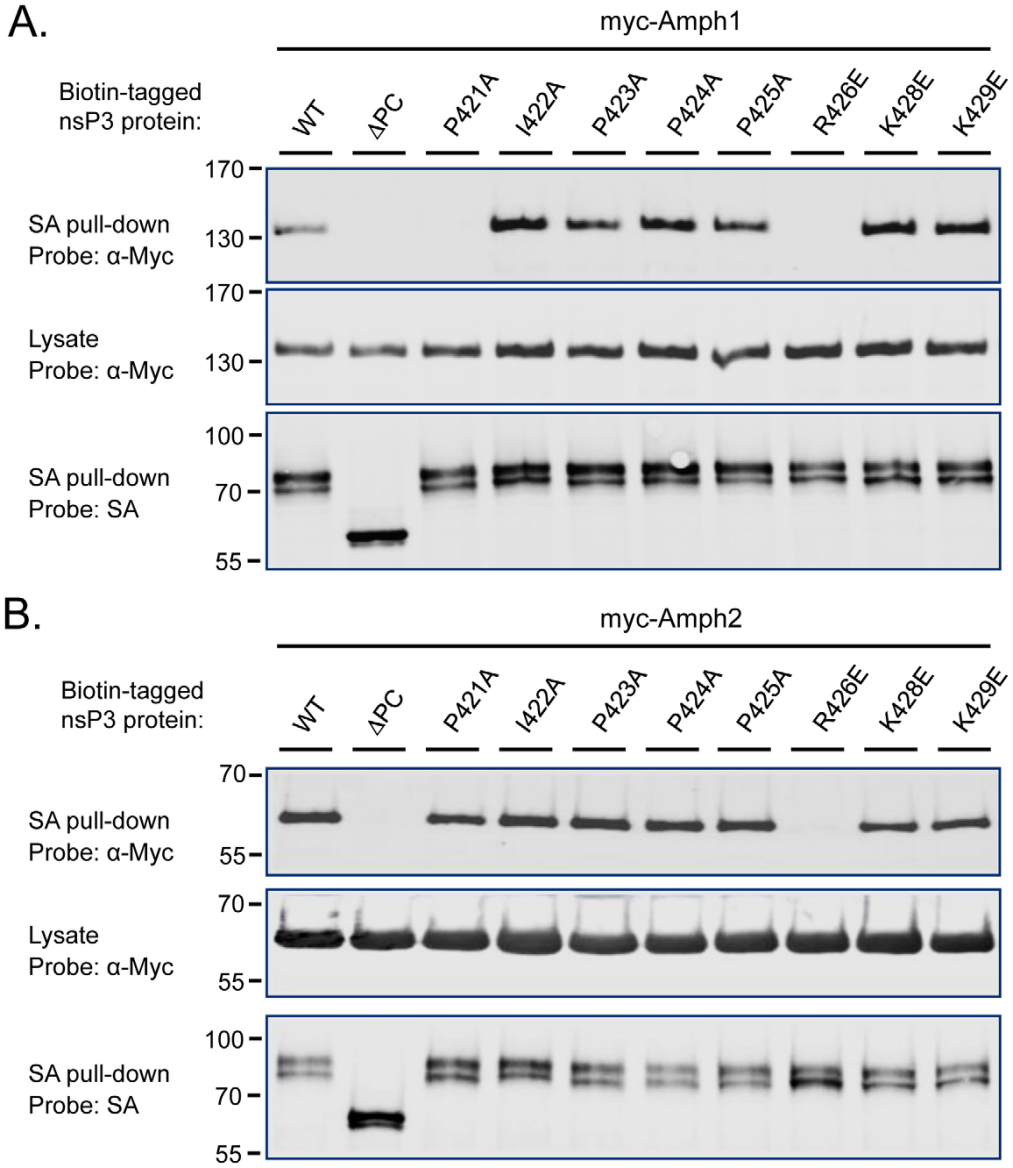
Identification of the critical amphiphysin-binding residues in the P1 region of SINV nsP3. Association of amphiphysin-1 (**A**) and amphiphysin-2 (**B**) with SINV nsP3 proteins carrying indicated single amino acid substitutions in the P1 region was examined as in Figure 2. The mutant indicated as ΔPC is lacking the complete P1 region plus the rest of the SINV nsP3 carboxyterminus.

As evident from Figures 2A and 2B (bottom panels) deletion of the P1 region in SINV nsP3 was associated with compromised stability of this protein. However, due to the complete loss of amphiphysin binding by this mutant (Figures 2A and 2B, top panels), as well the data on SINV nsP3 with overlapping mutations (ΔPC and R426E in Figure 3), the lower abundance of the SINV nsP3 ΔP1 mutant was unlikely explain its failure to associate with amphiphysins.

To confirm that an SH3-dependent interaction of nsP3 with an endogenous amphiphysin takes place during alphavirus infection we infected HeLa cells with wild-type SFV or a modified virus encoding the ΔP1+2 mutant of nsP3 (SFVΔP1+2). Western blotting detected similar amounts of wild-type and mutant nsP3 proteins in total cell lysates prepared from the infected cultures, as well as in anti-nsP3 immunoprecipitates of these lysates (Figure 2C, bottom and middle panels). By contrast, probing of the anti-nsP3 immunocomplexes with an anti-amphiphysin-2 antibody revealed a strong band of the expected size of 60 kDa from cells infected with wild-type SFV, whereas no evidence for amphiphysin co-precipitation with nsP3 could be seen from the cultures infected with SFVΔP1+2 (Figure 2C, top panel).

### Fine mapping of the amphiphysin SH3 binding site

To identify more precisely the nsP3 determinants responsible for amphiphysin SH3 binding we focused our attention to residues within the P1 region that were most conserved among SFV, SINV, and CHKV, as well as other alphaviral nsP3 proteins. Obvious candidates were the residues forming the sequence P(I/V)(P/A)PPR (“PIPPPR motif”) present in P1 region of the nsP3 proteins listed in Figure 1A. Mutations causing single amino acid substitutions in these or the adjacent conserved basic residues (K428 and K429) were introduced into SINV nsP3, and tested for their effects on binding to co-transfected amphiphysin-1 and -2 (Figure 3).

Although most of the individual amino acid changes were tolerated without a significant loss of binding in this co-expression assay, a charge-reversing change in the conserved arginine residue (R426E) of the PIPPPR motif completely abolished binding of SINV nsP3 to amphiphysin-1 (Figure 3A) as well as to amphiphysin-2 (Figure 3B). Binding to amphiphysin-1 was also largely abrogated by an alanine substitution of the first proline residue of the PIPPPR motif (P421A), whereas this mutation had less effect on amphiphysin-2 binding. This may reflect a real difference in the binding specificities of amphiphysin-1 and -2, but could also be at least in part due to the higher expression levels of the transfected amphiphysin-2. Due to the reduced stability of the ΔP1 mutant of SINV nsP3 (see Figure 2), as a negative control for amphiphysin binding we used here a larger deletion mutant of SINV nsP3 that in addition to the P1 region is lacking the carboxyterminal residues following this motif (ΔPC).

These studies further implicate the PIPPPR motif as a conserved amphiphysin docking site of alphaviral nsP3 proteins, and indicate the R426E mutation as a useful single amino acid substitution for creating an amphiphysin binding-defective derivative of SINV nsP3.

### Amphiphysin is recruited to alphavirus replication sites

To study the role of amphiphysin during the alphavirus replication cycle HeLa cells and N2A neuroblastoma cells were used as examples of cells that naturally express amphiphysin-2 or amphiphysin-1, respectively. The cells were infected with SFV or SINV and fixed at different time points during the infection, followed by staining with antibodies against nsP3, dsRNA (a marker for viral RNA to detect replication complexes), and amphiphysin-2 or amphiphysin-1.

**Figure 4:**
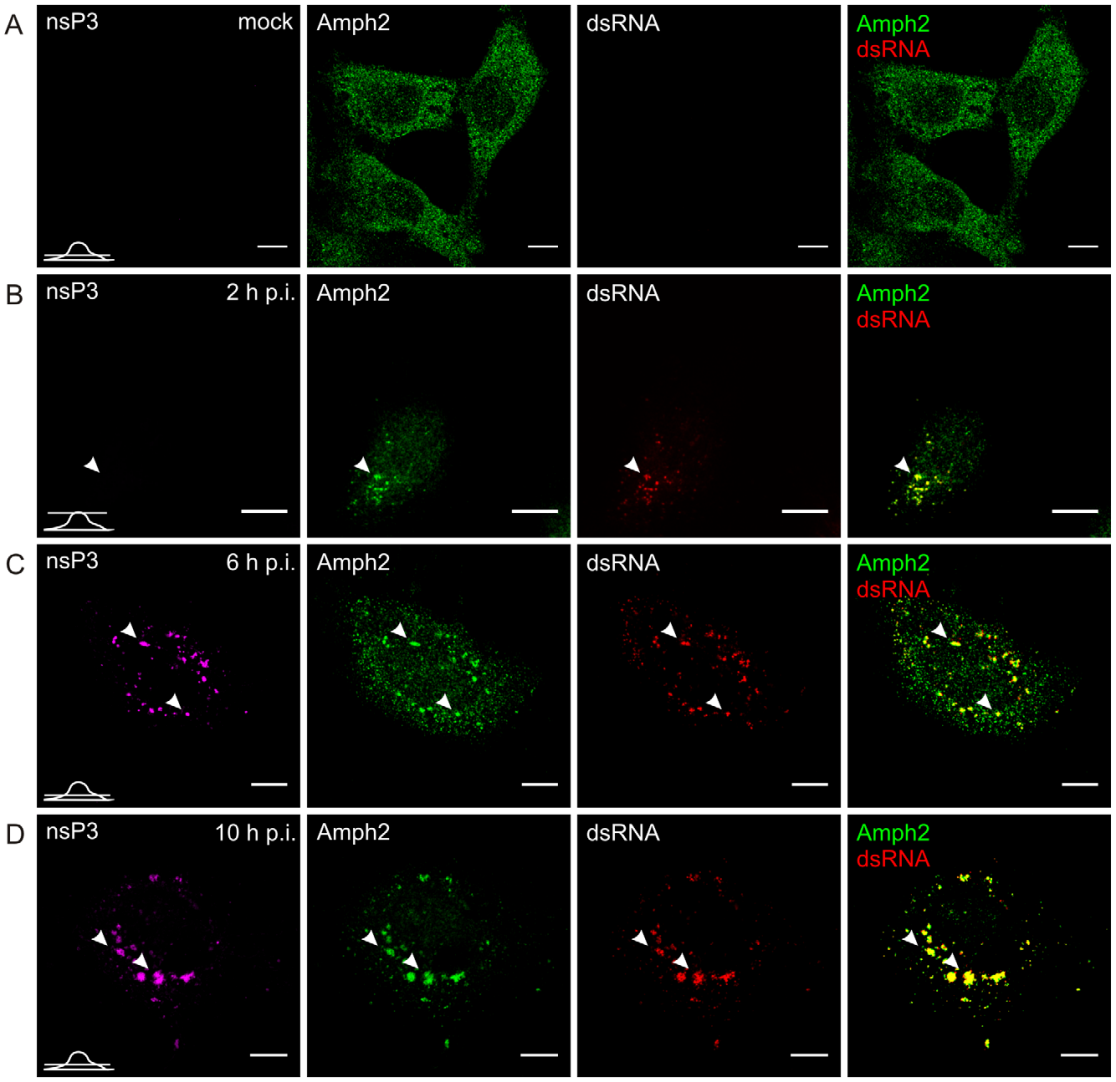
Recruitment of endogenous amphiphysin-2 to SFV replication sites in HeLa cells. Cells were infected with SFV at m.o.i. 50 (**B**, **C**, and **D**) or mock infected (**A**), fixed at the indicated time points and stained with specific antibodies against nsP3, amphiphysin-2, or dsRNA. Bound antibodies were detected with fluorophore-linked secondary antibodies, and the images were pseudocolored for visualization (nsP3 – magenta, dsRNA – red and amphiphysin – green). Each channel is shown in a separate image, and the overlay of dsRNA (indicating the localization of RCs) and amphiphysin-2 staining is shown on the right (colocalization seen in yellow). A representative image of the main phenotype detected is shown at each time point. Arrowheads: the localization of RCs matches with nsP3 staining. Single confocal sections are shown, and the position of each section is indicated in the bottom left corner of each row. The scale bars are 10 µm.

In uninfected HeLa cells amphiphysin-2 was localized in a diffusely distributed dot-like pattern throughout the cell (Figure 4A). When the cells were infected with SFV, colocalization of amphiphysin-2 and replication complexes could be detected already at 2 h post-infection (p.i.) when the replication complexes (RCs) started to form at the plasma membrane (Figure 4B). At 6 h p.i. distribution of amphiphysin-2 was markedly changed as a result of recruitment to RCs (Figure 4C). At late stage of the infection (10 h p.i.) when RCs were gathered to virus induced cytopathic vacuoles (CPVs) most of the cellular amphiphysin-2 localized to CPVs (Figure 4D; Figure S1A). Similar recruitment of amphiphysin-2 was also detected at later time points in HeLa cells when a lower multiplicity of infection (m.o.i.) of 1 was used, and in BHK cells in which amphiphysin-2 was recruited efficiently to CPVs (data not shown).

**Figure 5:**
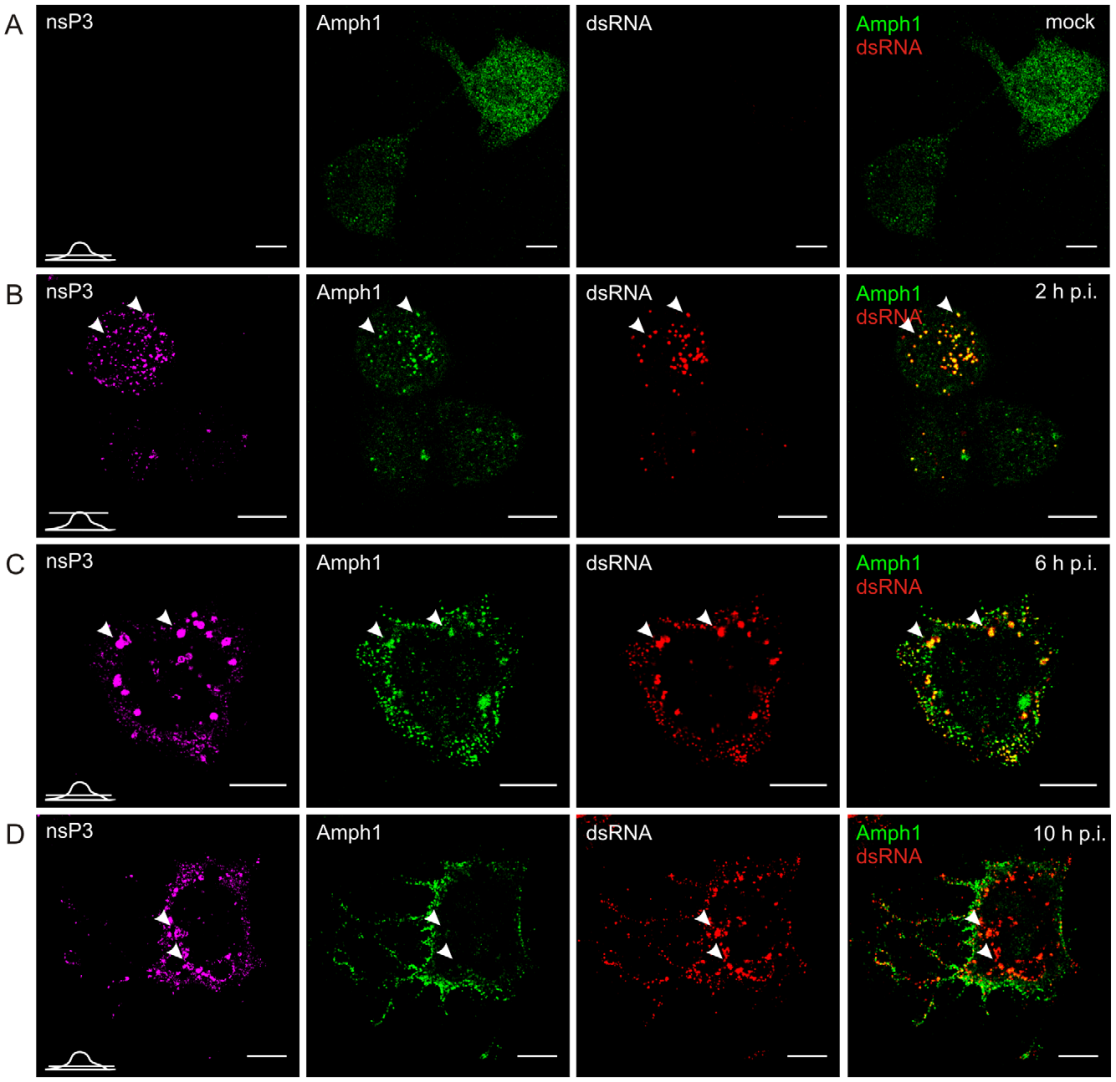
Dynamic changes in amphiphysin-1 localization during SFV infection of neuronal cells. N2A cells were infected with SFV (**B**, **C**, and **D**) or mock infected (**A**), and examined for nsP3, amphiphysin-1, or dsRNA staining at the indicated time points as in Figure 4.

In uninfected N2A cells amphiphysin-1 localization showed a similar diffuse pattern (Figure 5A) as observed for amphiphysin-2 in HeLa cells. Upon SFV infection amphiphysin-1 was first associated with RCs at 2 and 6 h p.i. (Figures 5B and 5C), again in a manner similar to amphiphysin-2 in HeLa cells. However at the very late stage of the infection (10 h p.i.) amphiphysin-1 was strongly relocalized to the plasma membrane and virus-induced filopodia/neurite structures (Figures 5D and S1B), thus differing from the pattern seen in HeLa cells where amphiphysin-2 remained associated with the replication complexes throughout the infection. This probably reflects different functions of amphiphysin-1 and amphiphysin-2, since amphiphysin-1 is thought mainly to function in synaptic vesicle endocytosis, whereas diverse cytoplasmic as well as nuclear functions not related to endocytic vesicle formation at the plasma membrane have been described for non-neuronal amphiphysin-2 isoforms [26], [27].

**Figure 6:**
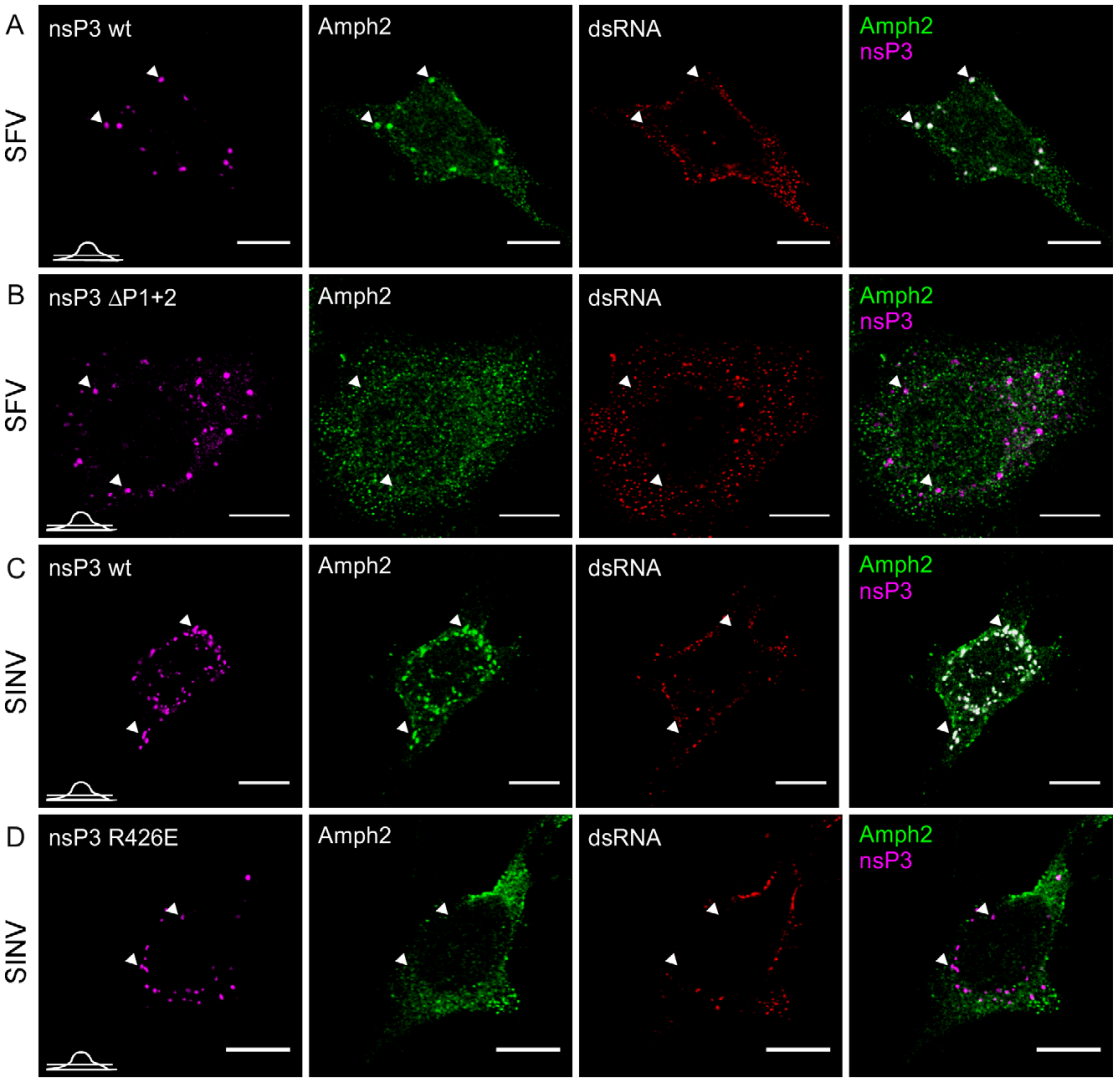
Amphiphysin is recruited by nsP3 in infected cells in an SH3-binding motif-dependent manner. HeLa cells were infected with wild-type SFV (**A**) or SINV (**C**) or the corresponding mutant viruses with SH3 binding motif-deficient nsP3 proteins, ΔP1+2 mutant of SFV nsP3 (**B**) and R426E mutant of SINV nsP3 (**D**) at m.o.i. 50, fixed at 10 h p.i. (6 h p.i. for wild-type SFV) and stained with specific antibodies for nsP3 (SFV), amphiphysin-2, or dsRNA. Bound antibodies were detected with fluorophore-linked secondary antibodies and the images were pseudocolored for visualization. SINV nsP3 was detected via its fluorescent fusion partner (pseudocolored in magenta). The overlay of nsP3 and amphiphysin-2 staining is shown on the right (colocalization seen in white). The images were chosen to show the phenotype representing nsP3 granules, which are present in a subpopulation of the cells. Arrowheads indicate nsP3 granules devoid of viral dsRNA. The position of each confocal section is indicated in the left bottom corner of each row. The scale bars are 10 µm.

As illustrated in Figure 4, and in agreement with earlier studies [15], in HeLa cells infected with SFV most of nsP3 was detected in dsRNA-positive vesicles that contained viral RCs, but a subpopulation of cells also contained large dsRNA-negative nsP3-containing granules which efficiently recruited amphiphysin (Figure 6A). A similar pattern was seen in cells infected with SINV (Figure 6C), which also showed nsP3-containing structures devoid of dsRNA in the cytoplasm. In contrast to SFV infection, in SINV-infected cells RCs were detected almost exclusively on the plasma membrane. Nevertheless, amphiphysin-2 similarly colocalized with SINV nsP3 both in the dsRNA-negative nsP3 structures as well as in the RCs (Figure 6C).

### Role of SH3 binding in amphiphysin recruitment in infected cells

Our experiments on the interactions between nsP3 and amphiphysin proteins (Figures 2 and 3) in co-transfected and in infected cells showed that proline-rich regions P1 and P2 contributed to the nsP3-amphiphysin interaction of SFV, whereas in SINV nsP3 this interaction was mediated by its sole SH3 binding motif (P1), and the positive charge of the SINV nsP3 residue R426 was found to be critical.

To examine if these SH3-mediated interactions were involved in the colocalization of amphiphysin and nsP3 proteins in the infected cells, we employed the SFVΔP1+2 virus used in Figure 2C, and also created a mutant of SINV expressing the SH3 binding-deficient nsP3 protein (SINV-R426E). To facilitate SINV nsP3 visualization the fluorescent protein Cherry was introduced in frame with nsP3 in SINV-R426E as well as in its wild-type counterpart. Such a modification has been previously used and shown to give rise to a virus that replicates with kinetics similar to a wild-type virus [28]. In contrast to cells infected with the corresponding wild-type SFV or SINV (Figures 6A and 6C) no amphiphysin-2 staining was observed in the dsRNA-negative nsP3-containing granules in cells infected with the mutant viruses, confirming this association to be strictly dependent on the SH3-mediated nsP3/amphiphysin interaction (Figures 6B and 6D).

**Figure 7:**
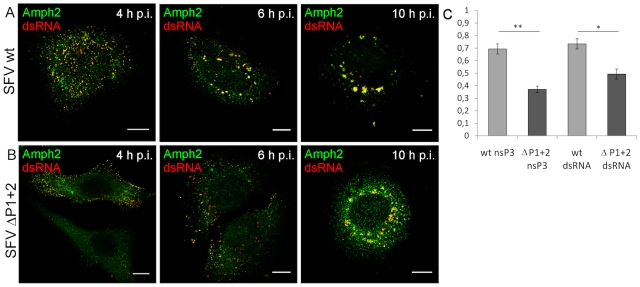
Deletion of the SH3 binding motif impairs amphiphysin-2 recruitment to RCs, and delays CPV formation. HeLa cells were infected with SFV wild-type (**A**) or SFV ΔP1+2 (**B**) at m.o.i. 50, fixed at the indicated time points and stained with specific antibodies. Colocalization of RCs (dsRNA staining) and amphiphysin-2 is shown in yellow. The scale bars are 10 µm. (**C**) Deletion of SH3 binding motif impaired amphiphysin-2 recruitment to replication complexes, as indicated by Pearson's coefficients measured for colocalization of amphiphysin-2 with dsRNA and nsP3 in CPVs at 10 h p.i. (n = 8-13 fields examined (at least 34 cells); *p<0.05; ** p<0.005).

Notable albeit less definitive differences between the wild-type and mutant viruses were also seen when amphiphysin-2 recruitment to viral RCs was examined (Figure 7, panels A and B, and data not shown). When HeLa cells were infected with wild-type SFV amphiphysin-2 colocalized strongly with nsP3 and dsRNA in CPVs at 10 h p.i. (Pearson's correlation coefficient (r) 0,69 and 0,73 respectively), whereas for ΔP1+2 virus the data analysis indicated only a weak colocalization (r_nsP3_  =  0,37; r_dsRNA_ =  0,49) (Figure 7C). Even at 12 h p.i. amphiphysin was diffusely localized in SFV ΔP1+2-infected cells and only faintly co-localized with RCs (data not shown). Moreover, as also evident from Figure 7 (compare panels A and B) a markedly delayed CPV formation in SFV ΔP1+2-infected cells was observed.

In conclusion, we found that infection with SFV or SINV encoding nsP3 proteins lacking SH3 binding capacity was clearly associated with impaired colocalization of amphiphysin-2 with nsP3 and RCs, as well as with reduced formation of SFV-induced CPVs.

### Amphiphysin binding by nsP3 is required for efficient viral RNA replication

To examine the relevance of the nsP3/amphiphysin-interaction for viral replication we used quantitative RT-PCR to compare viral RNA synthesis in HeLa cells as well as in BHK cells (a commonly used cell line for alphavirus propagation) infected with SFV and SINV encoding wild-type or SH3 binding-deficient nsP3 proteins. As the delay in CPV formation by SFVΔP1+2 virus was most obvious during the first 8 h of infection, we collected RNA specimens from the infected cells during this period.

**Figure 8:**
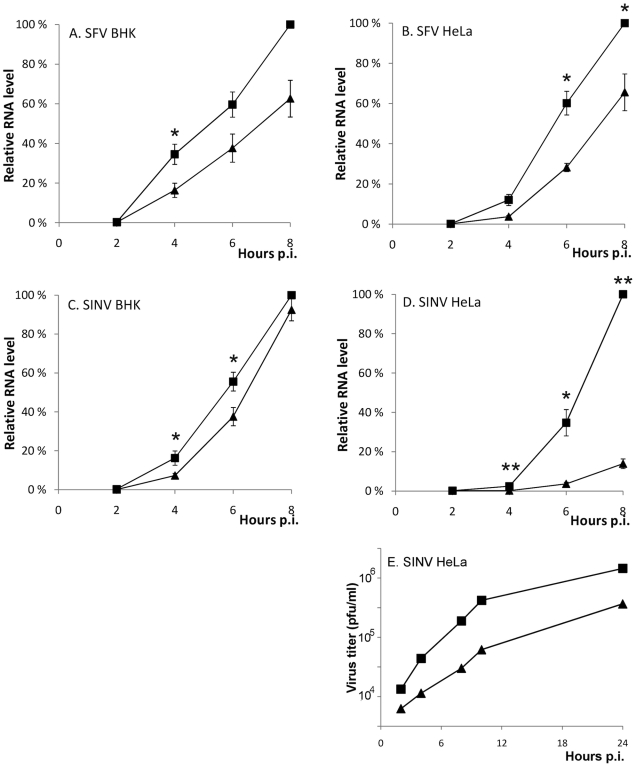
A functional SH3 binding motif in nsP3 is required for optimal SFV and SINV replication. (**A-D**) BHK and HeLa cells were infected with SFV (wild-type ▪ or ΔP1+2 ▴) and SIN (wild-type ▪ or R426E ▴) at m.o.i. 5. The total RNA was collected at 2 h intervals until 8 h p.i., and the viral RNA levels were detected by RT-qPCR. In each experiment wild-type 8 h sample was set as 100%, and the RNA amounts for each time point are shown relative to this. The measurements correspond to the mean value of three biological replicates. Standard deviations are indicated by error bars (n = 3; * P<0.05; ** P<0.001). (**E**) Aliquots of growth medium were withdrawn at indicated time points and the virus production was measured by plaque titration. The data shown are representative of two independent experiments.

The RNA synthesis of the SFVΔP1+2 was clearly reduced in both cell lines, as it produced ∼40% less RNA compared to wild-type SFV (Figure 8 A and B). A similar replication defect was seen for the SINV-R426E virus upon infection of the highly permissive BHK cells (Figure 8C). Interestingly, a striking difference in the replicative capacity of SINV encoding for an SH3 binding-competent or -defective nsP3 was observed in HeLa cells, where SINV-R426E RNA synthesis was found to be severely impaired (Figure 8D). In agreement with the decreased viral RNA levels, reduced production of infectious virus particles by the mutant SINV virus was also observed (Figure 8E).

**Figure 9:**
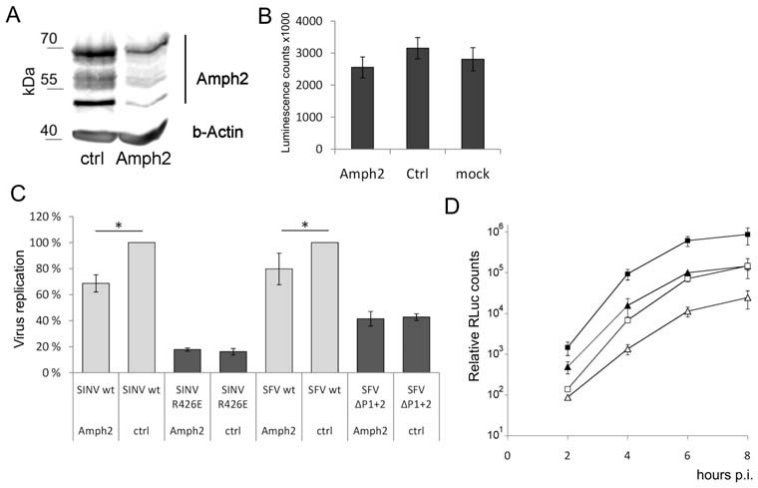
Amphiphysin silencing impairs viral RNA replication. (**A**) HeLa cells were treated for 68 h with amphiphysin-2 or control siRNAs. Levels of amphiphysin-2 isoforms expressed were detected by Western blot. Beta-actin staining is shown as a loading control. (**B**) Viability of the siRNA-transfected cells was analyzed by measuring the cellular ATP levels using a luminescence based assay. Cell cultures that were left untreated are included for comparison (n = 3). (**C**) Cells treated with siRNA were infected in parallel with wild-type or mutant viruses at m.o.i. 5, and RNA replication was measured at 5 h p.i. by RT-qPCR (n = 3; * p<0.05). (**D**) Cells were infected with SFV-RLuc at m.o.i. 0.5 (□ control Δ amphiphysin-2 siRNA) or m.o.i 5 (▪ control ▴ amphiphysin-2 siRNA), and viral replication was assayed based on Renilla luciferase activity (RLuc) at the indicated time points. The data shown represent the average of two independent experiments.

To confirm that the observed reduction in viral RNA replication was indeed amphiphysin dependent, we knocked down amphiphysin-2 expression in HeLa cells via RNA silencing Several approaches to this end were utilized. Figure 9 shows data obtained using a pool of siRNAs validated for amphiphysin-2 knockdown by Dharmacon. Results in good agreement with these were also obtained using an unrelated set of non-overlapping siRNAs obtained from Qiagen, as well as by studying a panel of HeLa cell clones stably transduced with different amphiphysin-2-specific shRNA-expressing lentiviruses (data not shown). Although a complete loss of amphiphysin-2 protein expression could not be achieved by any of these approaches, as shown in Figure 9A, a three-day treatment of HeLa cells with the pool of Dharmacon siRNAs consistently led to a strong suppression of amphiphysin-2 expression. This knockdown treatment was not associated with noticeable cytotoxicity, as no indication of reduced metabolic activity was observed using a commercial cell viability assay (Figure 9B).

As shown in Figure 9C (grey bars), the suppression of amphiphysin-2 expression was associated with a clearly reduced capacity of wild-type SINV and SFV to replicate in these cells. Although this reduction was not as severe as that observed for the SH3 binding site-defective mutants, it was statistically significant. A plausible explanation for the lower inhibitory effect of amphiphysin-2 knockdown may be the residual levels of amphiphysin-2 remaining in the knockdown cells (Figure 9A). Of note, the amphiphysin knockdown did not have any negative effect on the residual replication of the SH3 binding site-defective mutant viruses (Figure 9C, dark bars), thus excluding the contribution of indirect effects of amphiphysin depletion that might not be mediated via nsP3 binding, as well as toxic or off-target effects of the siRNA treatment.

To examine in more detail the effect of amphiphysin-2 knockdown HeLa cells were infected with a luciferase marker-expressing virus SFV-RLuc using two different m.o.i (0.5 and 5), and the replication of this virus was monitored at several time-points after infection based on the luciferase activity in the infected cultures pretreated with amphiphysin-2 or control siRNAs. As shown in Figure 9D, irrespective of the viral inoculum SFV replication was suppressed at all time-points by amphiphysin-2 knockdown. The degree of suppression of SFV RNA replication was consistently even greater (over 5-fold) when determined using this reporter system as compared to direct measurement using RT-qPCR. However, both approaches showed that suppression of amphiphysin-2 expression, even if partial, consistently led to a statistically significant suppression in viral replication.

### SFV carrying amphiphysin binding-defective nsP3 is attenuated in mice

**Figure 10:**
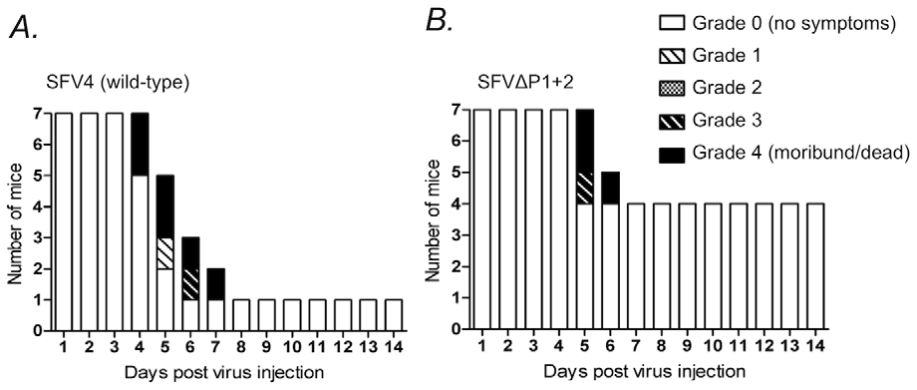
Neurologic symptoms and mortality in Balb/c mice following SFV infection. Female 6-week-old Balb/c mice were infected i.p. with 1×10^6^ pfu of (**A**) SFV4 or (**B**) SFVΔP1+2 mutant virus. Bars indicate the number of surviving mice, and grading of the clinical status of the mice at each day during a period of two weeks after the infection. The complete grading system used was: 0  =  no symptoms; 1  =  weakness of limbs, or hunched back, or ruffled fur, or any combinations of these; 2  =  partial paralysis of hind limbs; 3  =  paralysis of limbs, limited moving or abnormal moving behavior; 4  =  moribund or dead.

To further address the biological significance of the decreased replicative capacity of the nsP3-mutated viruses, we injected the SFVΔP1+2 virus intraperitoneally into Balb/c mice, and compared the neurologic symptoms and survival of mice infected with the parental wild-type (neurovirulent) SFV4 virus. Only one out of seven of SFV4-infected mice survived and remained asymptomatic, whereas the residual six mice in this group all developed severe paralytic symptoms 4 to 7 days post infection and were found dead or were sacrificed (Figure 10A). By contrast, in the group of mice infected with SFVΔP1+2 four mice out of seven remained completely asymptomatic 14 days post infection (Figure 10B). It is interesting, however, that all SFVΔP1+2 infected mice that initially displayed neurological signs later died or became moribund and were euthanized, as also seen in SFV4-infected mice. This suggests that the defect in SFVΔP1+2 replication in mice may be mainly manifested early in the course of the infection before the virus enters the brain. In any case, these results revealed a significant attenuation of the SFVΔP1+2 in vivo resulting in reduced pathogenicity in infected mice.

## Discussion

In this study we have shown that despite their striking lack of overall sequence homology the C-terminal regions of nsP3 proteins encoded by several alphaviruses contain a conserved SH3 binding motif (dubbed the PIPPPR motif) that can recruit the SH3 domain of amphiphysin-1 and -2. These two related proteins are members of the BAR (Bin-Amphiphysin-Rvsp) protein superfamily implicated in several cellular functions, of which the key role of amphiphysin-1 in clathrin-mediated endocytosis of synaptic vesicles has been studied most extensively [27], [29], [30].

PIPPPR motif-containing nsP3 proteins from three alphaviruses, namely SFV, SINV, and CHKV were included in our study, and shown to interact avidly and in an SH3 binding motif-dependent manner with amphiphysin-1 and -2 in biochemical as well as in cell-based experimental systems. In addition to the PIPPPR motif SFV nsP3 was found to contain another proline-rich region that could also support some binding to amphiphysin SH3. Since the C-termini of all alphaviral nsP3 proteins include proline-rich regions that might serve as amphiphysin SH3 binding sites, it is possible that the capacity to interact with amphiphysin is shared also by alphaviral nsP3 proteins that do not contain an obvious PIPPPR motif. This possibility is currently under investigation.

Insects have only a single amphiphysin gene [31], which is very similar in its structure and organization with mammalian amphiphysin-1 and -2, and encodes a homologous SH3 domain differing mainly in the distal loop, a region that is not involved in SH3 ligand binding [32]. In experiments not included in this paper we have confirmed binding of the *Drosophila melanogaster* amphiphysin SH3 to SFV, SINV, and CHKV nsP3 proteins (AK, unpublished observations). This SH3 domain is almost identical to those of the mosquitos *Aedes aegypti* and *Anopheles gambiae*, suggesting that the nsP3 interactions with human amphiphysins examined here may be relevant also during alphaviral replication in their insect hosts.

Studies using infectious SFV and SINV confirmed SH3-mediated binding of virally produced nsP3 with endogenous amphiphysin-1 and -2 in neuronal and in non-neuronal cells, respectively. These interactions were associated with major changes in the intracellular localization of amphiphysin in the infected cells. Recruitment of amphiphysin to nsP3-containing viral dsRNA-negative cellular granules was completely dependent on the SH3 binding site of nsP3. A robust amphiphysin relocalization to SFV and SINV replication complexes (RCs) that stained positive for both nsP3 and dsRNA was also observed, and was clearly enhanced by the amphiphysin SH3 binding capacity of nsP3. However, recruitment of amphiphysin to RCs was also seen upon infection with viruses encoding nsP3s that lacked the SH3 binding site and failed to physically interact with amphiphysin-1 or -2 in our co-precipitation assays. We hypothesize that in addition to the SH3 domain-mediated targeting to the RCs via nsP3, amphiphysin might also be recruited to these structures via its BAR domain, which functions in deforming membrane architecture and sensing of the membrane curvature [33], [34]. Alphavirus RCs are housed in specialized virus-induced membrane invaginations called spherules [5], [35]. Although the body of such a spherule poses a negative curvature towards the cytoplasm, the neck region of a spherule presents a positive membrane curvature with a diameter of 10-15 nm that is compatible with the binding characteristics of N-BAR domains [26], [35]. Thus, it is easy to envision that the membranes associated with RCs might attract amphiphysin to these structures in a BAR domain-dependent but SH3 domain-independent manner. The amphiphysin BAR domain might be actively involved in the formation or stabilization of the RC-associated membrane structures.

We have attempted to study the role of RC-recruitment of amphiphysin-2 by electron microscopy (EM), but have not noted obvious defects in spherule morphology upon infection with SFV carrying an SH3-binding deficient nsP3 or upon infection of amphiphysin-2 siRNA treated HeLa cells with wild-type SFV (data not shown). Although not statistically confirmed due to limitations inherent to EM, such manipulations do seem to be associated with reduced spherule formation.

Mutational analysis of the nsP3 PIPPPR motif revealed the arginine residue of this sequence as the most critical amino acid in amphiphysin SH3 domain binding. Interestingly, the PIPPPR sequence complies better with the classical type II consensus SH3 binding motif PxxPxR rather than the atypical motif PxRPxR proposed as the optimal amphiphysin SH3 recognition sequence [36]. However, due to the unusual architecture of the proline-binding groove of the amphiphysin-1 and -2 SH3 domains [37], [38], canonical type II docking of the nsP3 PIPPPR peptide may not be readily assumed. Further structural studies will be needed to establish on the molecular level how the amphiphysin SH3 domains accommodate alphaviral nsP3 proteins.

When introduced into the viral genome mutations disrupting the amphiphysin SH3 binding sites in SFV or SINV nsP3 resulted in impaired viral RNA synthesis, thus revealing a positive role for the nsP3/amphiphysin interaction in alphaviral replication. The defect in replication was especially pronounced when RNA synthesis of wild-type SINV was compared to SINV-R426E in HeLa cells. Thus, it is possible that the relative requirement for nsP3-mediated amphiphysin recruitment depends on factors such as permissivity of a particular cell-type for replication of the alphavirus in question.

It is remarkable that a single point-mutation in the C-terminal “non-conserved” region of SINV nsP3 was capable of causing such a pronounced defect in RNA synthesis, since it has been previously described that in general even large deletions in this region are relatively well tolerated by SINV and SFV [12], [39], [40]. This comparison further highlights the functional importance of the SH3 binding sites described here.

While our affinity screening of a comprehensive human SH3 phage library clearly indicated amphiphysin-1 and -2 as the superior binding partners for SFV, SINV, and CHKV nsP3 proteins, it could not be ruled out that other host cell SH3-containing proteins could be engaged in functionally significant interactions with nsP3 despite having low intrinsic binding affinity. However, our results on the effects of specific silencing of amphiphysin-2 in HeLa cells on the replication kinetics of SFV and SINV strongly support the idea that amphiphysins are not only the preferred interaction partners of nsP3, but also account for the enhanced replication of the wild-type viruses as compared to the SFVΔP1+2 and SINV-R426E mutants.

The significance of the replication defect in cell culture of the SH3 binding site-defective viruses was supported by a clear phenotype observed in infected mice. Neurological symptoms and mortality of mice infected intraperitoneally with SFVΔP1+2 were greatly reduced compared to wild-type SFV. Further studies in this mouse model are clearly warranted to develop a better understanding of the biological consequences of the disrupted nsP3/amphiphysin interaction, including the specific tissue(s) where replication of the mutant virus would be most compromised.

Further clarification of the mechanistic basis of the positive effect on alphavirus replication provided by nsP3-mediated amphiphysin recruitment poses an important and interesting challenge for future investigations. In this regard, it is worth noting that a better understanding of the cellular processes involved might have more general implications on virus-host cell interactions beyond alphavirus biology. Mass spectroscopic identification of cellular partners of the hepatitis C virus (HCV) non-structural protein 5A (NS5A) revealed amphiphysin-2 as an NS5A-binding protein, and this interaction was subsequently mapped to the SH3 domain of amphiphysin-2 and a proline-rich region in NS5A [41], [42]. Strikingly, the sequence of NS5A in this region shows extensive homology with the P1 region of alphaviral nsP3 proteins, including a bona fide PIPPPR motif, thus defining this non-canonical amphiphysin SH3 target site as a general viral amphiphysin interaction motif.

Unlike in the current case of SFV and SINV, mutations affecting the proline-rich region in NS5A did not have an obvious effect on viral replication, at least as judged by the use of the subgenomic replicon model system for HCV infection in cultured Huh-7 cells [42]. However, usurping amphiphysin via a similar strategy, together with the replication defect caused by disruption of this interaction now observed for alphaviruses should encourage further efforts for addressing the significance of the amphiphysin/NS5A complex for HCV infection and pathogenesis. As a member of the family Flaviviridae HCV is not related to alphaviruses. Nevertheless, similar to other positive-strand RNA viruses, HCV replication is associated with extensive reorganization of cellular membranes, and takes place in specialized NS5A-containing membrane structures that in the HCV literature are referred to as membraneous web [43], [44]. More generally speaking, the intriguing parallels discussed above suggest that amphiphysin might play some ubiquitous role in host cell membrane rearrangements characteristic of many RNA viruses, and may deserve attention as a host cell factor with potential in development of future antiviral strategies.

## Materials and Methods

### Cell culture

HeLa and N2A cells were cultured in Dulbecco's modified Eagle's medium (DMEM) supplemented with 10% inactivated fetal bovine serum (FBS) (Gibco-BRL, Grand Island, NY), 2 mM L-glutamine, 100 U/ml penicillin, and 100 µg/ml streptomycin (Gibco). BHK cells were cultured in DMEM supplemented with 7,5% inactivated FBS, 2% tryptose phosphate broth, 2 mM L-glutamine, 100 U/ml penicillin, and 100 µg/ml streptomycin (Gibco).

### Plasmid constructs and viruses

pEBB/PP-SFVnsP3, pEBB/PP-SINVnsP3, and pEBB/PP-CHKVnsP3 plasmids encoding SFV, SINV, and CHKV nsP3 fusions with the biotin acceptor domain were constructed by subcloning the corresponding nsP3-encoding PCR products to pEBB/PP vector [20], [21] by using EcoRV-NotI, StuI-NotI, and KpnI-NotI restriction sites, respectively. These constructs were further used as templates for PCR to generate proline-rich region-deletion mutants of nsP3. For generation of expression vectors encoding different point-mutants of SINV nsP3, pEBB/PP-SINVnsP3 was used as a template for PCR with a set of point-mutagenesis primers. For expression of the epitope-tagged amphiphysin-1 and -2 the IMAGE cDNA clones corresponding to GenBank accession numbers BC034376 and BC004101, respectively, were inserted in frame with the Myc-tag into the polylinker of the pCMV-Myc plasmid (Clontech).

Genomic SFV construct, pSFVΔP1+2 was made in three steps. First, an intermediate construct, pEBB/SFV4SacNot, was generated by subcloning SacI-NotI fragment from SFV4 genome DNA [45] into pEBB vector opened with SacI+NotI. This construct was used as a template for a subsequent deletion of the P1+2 region by PCR, which resulted into the plasmid pEBB/SFV4SacNotΔP1+2. In the final step, SacI-NotI fragment from pEBB/SFV4SacNotΔP1+2 was subcloned into SFV4 opened with SacI+NotI. Genomic SINV construct, pSINV-R426E carrying R426E mutation of nsP3 was constructed in three steps. First, BamHI-BamHI fragment from pToto/1101 [46] was transferred to pEBB vector opened with BamHI, resulting in an intermediate construct pEBB/SINVBB. This construct was subsequently used as a template for introducing the mutation R426E into nsP3 using PCR with mutated primers. In the final step, BamHI-BamHI fragment from pEBB/SINVBB was ligated back to the BamHI restriction site of pToto/1101. To generate pSINV-Cherry and pSINV-R426E-Cherry, mCherry-encoding DNA fragment, generated by PCR and digested with SpeI, was inserted into SpeI site of pToto/1101 and pSINV-R426E, respectively. To generate infectious viruses RNA was transcribed from these vectors and viruses were producted in BHK cells as described previously [47].

### Phage display

Panning of the SH3 phage display library using target proteins was performed as described earlier [19]. Briefly, biotin-tagged nsP3 proteins were expressed in 293FT cells and precipitated with streptavidin-coated magnetic beads from lysates as described below in “Protein pull-downs and Western blots”. The precipitated material was incubated with the mixture of human SH3 library-displaying phages (10^9^–10^10^ colony forming units (cfu) per well), prepared in PBS-T (0.05% Tween-20 in 1xPBS) and supplemented with 2.5% of non-fat milk, for 2 h at room temperature. The non-bound phages were then removed and the beads were washed 4 times with 1 ml of PBS-T. Subsequently, the nsP3 bound phages were incubated with TG1 bacteria (grown at the log-phase of OD600 = 0.5-0.6) at 37°C for 1 h and the infected bacteria seeded onto ampicillin-containing LB plates. NsP3-interacting SH3 domains were identified by sequencing of SH3 domain-encoding phagemides (pG8J8.SH3 clones [19]) obtained from individual bacterial colonies.

### Antibodies

Polyclonal nsP3 antibodies from rabbit and guinea pig have been described previously [5]. Mouse monoclonal antibody J2 against double-stranded RNA was purchased from Scicons (Hungary). Rabbit polyclonal antibody H100 against amphiphysin-2, mouse monoclonal antibody 2F11 against amphiphysin-2, and goat polyclonal antibody N19 against amphiphysin-1 were from Santa Cruz Biotechnology. Secondary antibodies were conjugated with Alexa-488, Alexa-568 and Alexa-647 (New England Biolabs) or Cy5 (Abcam). Mouse anti-Myc antibody M-5546 was from Sigma, infrared fluorescence dye IRDye680-labeled goat anti-mouse antibody and IRDye 800CW-streptavidin were from LI-COR Biosciences.

### Immunofluorescence and confocal microscopy

HeLa and N2A cells were infected with SINV Toto1101 or SFV4 (wild-type or mutant) with an m.o.i. of 50. For indirect immunofluorescence, cells were fixed at indicated time points, at room temperature with 4% paraformaldehyde in phosphate-buffered saline for 20 min, followed by quenching with 50 mM NH_4_Cl, and permeabilized with 0.1% Triton X-100. Coverslips were incubated with primary and secondary antibodies and mounted on Mowiol 4-88 (Calbiochem) containing 2.5% DABCO (1,4-diazabicyclo(2,2,2) octane; Sigma). Images were obtained with Leica TCS SP5 upright confocal microscope using an HCX APO 63x/1.30 numerical aperture, corrected for 21°C glycerol objective.

### Image analysis and colocalization studies

Immunofluorescence images were processed by using ImageJ (National Institutes of Health, Bethesda, MD). Images were pseudocolored so that amphiphysin-1 and 2 are presented in green, and nsP3 and dsRNA in magenta (blue in 3D) and red respectively. For 3D and colocalization analysis images were deconvoluted with Autoquant X 2.2.0 (AutoQuant Imaging, Inc.) and processed with Bitplane Imaris 7.1.1. colocalization software (Bitplane Inc.). For colocalization assessment 35 cells from each sample were analyzed with Imaris. The mean values and standard error of the mean (SEM) were calculated for the obtained Pearson's correlation coefficients.

### siRNA experiments

HeLa cells were transfected with siRNAs at 20 nM concentration using Oligofectamine Transfection Reagent (Invitrogen) according to the manufacturer's instructions. Amphiphysin2 was silenced using either Dharmacon ON-TARGETplus smart pool L-008246-00-0005 (sequences: 5′-GACAUCAAGUCACGCAUUG-3′; 5′-GAACAGCCGCG UAGGUUUC-3′; 5′-ACAACGACCUGCUGUGGAU-3′; 5′-CCAGCAACGUGCA GAAGAA-3′) or Qiagen FlexiTube GeneSolution for BIN1 Hs_Bin1_5 5′-CCGGCGGAATTCACCAGTGTT-3′; Hs_Bin1_6 5′CTGGTCGGCCTGGAGAAGCAA-3′; Hs_Bin1_2 5′ATGGCAGAGATGGGCAGTAAA-3′; Hs_Bin1_3 5′-CAAGCTCAA CCAGAACCTCAA-3′. As non-specific siRNA controls ONTARGETplus Non-Targeting Pool or Qiagen Negative Control siRNA were used. Cells were incubated for 68 h and infected using a m.o.i. of 5. For luciferase measurements SFV-RLuc virus [48] was used. Cells were lysed at 5 h p.i. either with Trizol-reagent (Invitrogen) or Renilla Luciferase Assay Lysis Buffer (Promega), and analyzed with RT-qPCR or luciferase assay, respectively. Luciferase measurements were conducted as described earlier [48]. For Western blot, cells were lysed at 68 h post transfection in Laemmli buffer. Viability of siRNA treated cells was determined by measuring the cellular ATP levels with a CellTiter-Glo Luminescent Cell Viability Assay (Promega).

### Quantitative PCR

BHK cells were infected with SFV and SIN with 5 plaque forming units (pfu) per cell. Cells were lysed with Trizol-reagent (Invitrogen) at 2, 4, 6 and 8 h p.i. for total RNA isolation. From each sample 200 ng of total RNA was reverse transcribed to cDNA by using High Capacity cDNA Reverse Transcription Kit according to manufacturer's instructions (Applied Biosystems). The cDNAs were diluted 1∶10 and quantitative PCR was run using LightCycler 480 SYBR Green I Master Mix (Roche) with virus RNA specific primers and primers for guinea pig glycerylaldehyde-3-phosphate dehydrogenase (GAPDH). The primer sequences were: SFV nsP1 forward: TCTTTGCAGAAGGCATTTCC, SFV nsP1 reverse: GCATGGTCATTTGGTGTGAC, SIN nsP1 forward: GAATGTTTTCCGAGCACCAG, SIN nsP1 reverse: CCGGGTCTTCTGGACTACG, BHK GAPDH forward: ATCCCACCAACATCAAATGG, BHK GAPDH reverse: AAGACGCCAGTAGACTCCACA, HeLa GAPDH forward: AGCCACATCGCTCAGACAC, HeLa GAPDH reverse: GCCCAATACGACCAAATCC. The relative levels of viral RNA were determined by using GAPDH as an endogenous control. Wild-type 8h sample was set as 100% and all the other samples were normalized against that.

### Growth curves

HeLa cells were infected with SINV-Cherry or SINV-R426E-Cherry at m.o.i. 5. Aliquots of growth media were withdrawn at 2 h intervals and the samples were analyzed in BHK cells by plaque titration as described earlier [49].

### Protein pull-downs and Western blots

For co-precipitation of transiently expressed proteins, 293FT cells were transfected by standard calcium phosphate precipitation method with expression vectors encoding different nsP3 proteins tagged with the biotin acceptor domain together with an equimolar amount of a vector for Myc epitope-tagged amphiphysin-1 (Gen Bank accession BC034376) or amphiphysin-2 (ubiquitous isoform #9; Gen Bank accession BC004101), corresponding to a total of 10–16 µg of plasmid DNA per 10 cm culture dish. After 24 h of transfection, cells were collected in PBG buffer (1x phosphate buffered saline (PBS) supplemented with 10% glycerol and 0.5% Tween-20) containing protease inhibitors (“Complete”, Roche) and lysed by sonication at 0.2–0.3 kJ on ice by Bandelin Sonoplus homogenizer. NsP3/amphiphysin complexes were precipitated using streptavidin-coated magnetic beads (Dynabeads M-280-Streptavidin, Invitrogen). Whole-cell extracts (WCEs) and precipitated material were analyzed by Western blotting, where mouse anti-Myc antibody together with IRDye-labeled secondary anti-mouse antibody were used for detection of amphiphysins and the IRDye-labeled streptavidin was used for detection of nsP3 proteins by Odyssey infrared imaging system (LI-COR Biosciences).

NsP3 interaction with endogenous amphiphysin-2 in SFV-infected HeLa cells was analyzed as follows. NsP3 proteins were precipitated from WCEs with guinea pig anti-nsP3 antibody, which was immobilized onto Protein-A-Sepharose resin (Invitrogen). Immunoblotting of protein complexes was carried out using rabbit antibody against amphiphysin-2 or rabbit antibody against nsP3 in combination with horseradish peroxidase-(HRP)-conjugated secondary anti-rabbit antibodies. Immobilon Western chemiluminescent HRP substrate (Millipore) was used for the detection.

In all co-precipitation experiments 2% aliquots of the unprocessed lysate were loaded into the gel to examine the abundance of vector- or virus-encoded proteins in the total lysate. The rest of the lysates were subjected to immuno/affinity-precipitation, and a third of these precipitates were loaded in the gel to examine the specifically precipitated and co-precipitated proteins.

### Statistical analysis

Data are reported as mean ± standard deviation, if not otherwise stated. Statistical analysis was performed using Microsoft Excel Students t-test.

### Viruses, infections of mice and clinical grading

SFV4 and SFVΔP1+2 with targeted deletions in proline-rich domain of nsP3 were prepared as described above. Groups of 6-week old Balb/c-mice (n = 7) were administered intraperitoneally 1×10^6^ plaque pfu of virus in 100 µl PBS. Mice were housed in day-night balanced rooms and observed daily for neurologic symptoms for 14 days and sacrificed in the case of severe distress or significant loss of weight.

### Ethics statement

The animal experiments have been approved by the Finnish Committee for Care and Use of Animals in Experiments with validated competence of the authors to perform all the experiments described in the study protocol under the licence number ESLH-2008-072321-Ym-23. The animal experiments have been be carried out in the Central Animal Laboratory of the University of Eastern Finland according to the European Convention for the Protection of Vertebrate Animals used for Experimental and other Scientific Purposes and The Law and the Statute on Animal Experiments in Finland (62/2006; 36/2006) and the EU Directive 2010/63/EU, the Statute 1076/85 § 3 and 1360/90.

## Supporting Information

Figure S1 Amphiphysin-1 is relocated to plasma membrane late in infection, whereas amphiphysin-2 remains attached to virus-induced CPVs in the perinuclear area (10 h p.i., m.o.i. 500). 3D models of SFV infected HeLa (**A**) and N2A (**B**) cells were produced with Imaris Bitplane program after deconvolution with Autoquant X.
(TIF)
