# Supplementary figures and images for: SH3 Domain-Mediated Recruitment of Host Cell Amphiphysins by Alphavirus nsP3 Promotes Viral RNA Replication
Source: PLoS Pathog. 2011 Nov 17;7(11):e1002383. doi: 10.1371/journal.ppat.1002383 (PMC3219718; doi:10.1371/journal.ppat.1002383)

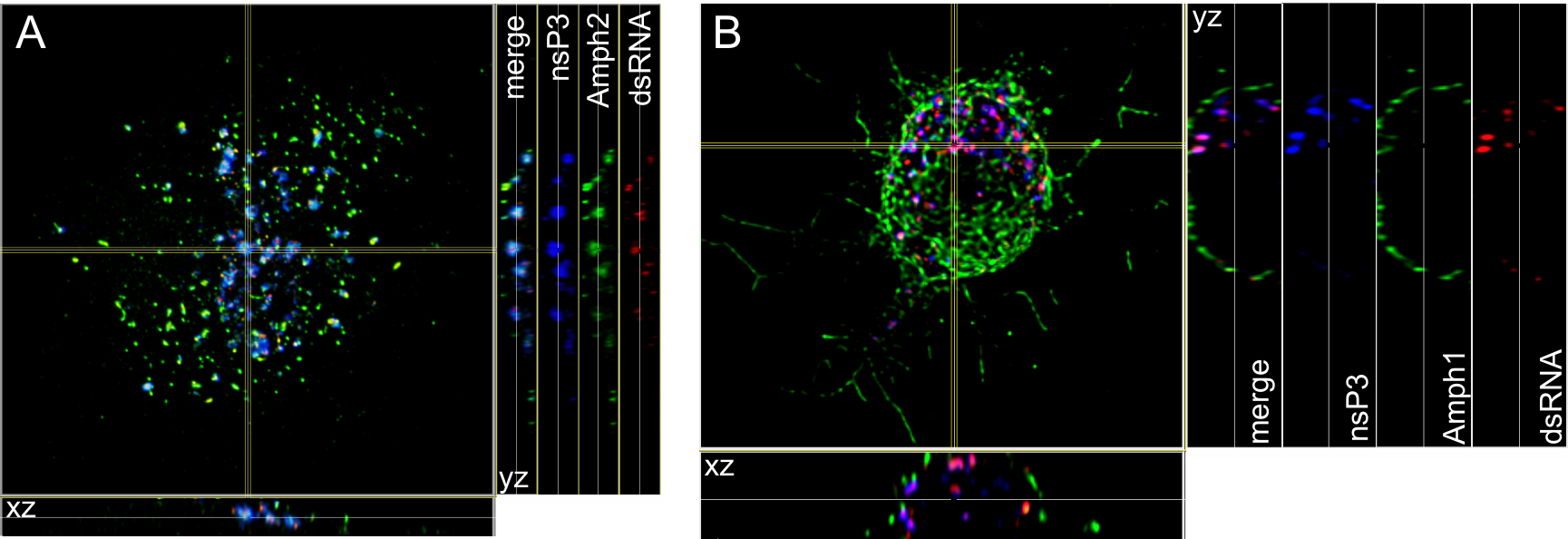

Supplement: Figure S1 — Amphiphysin-1 is relocated to plasma membrane late in infection, whereas amphiphysin-2 remains attached to virus-induced CPVs in the perinuclear area (10 h p.i., m.o.i. 500). 3D models of SFV infected HeLa (A) and N2A (B) cells were produced with Imaris Bitplane program after deconvolution with Autoquant X. (TIF) [file ppat.1002383.s001.tif]
